# Harmine activates intrinsic and extrinsic pathways of apoptosis in B16F-10 melanoma

**DOI:** 10.1186/1749-8546-6-11

**Published:** 2011-03-23

**Authors:** Thayele Purayil Hamsa, Girija Kuttan

**Affiliations:** 1 Amala Cancer Research Centre, Amala Nagar, Thrissur, Kerala, India, 680555

## Abstract

**Background:**

Harmine is a beta-carboline alkaloid from the plant *Peganum harmala*. Previous studies found that harmine inhibited metastasis of B16F-10 melanoma cells. This study aims to elucidate the role of harmine in apoptosis of B16F-10 cells.

**Methods:**

B16F-10 melanoma cells were treated in the presence and absence of harmine *in vitro*. Morphological changes, cell cycle and expression of various pro and anti- apoptotic genes were analyzed for the study of apoptosis.

**Results:**

Morphological observation and DNA laddering assay showed that harmine treated cells displayed marked apoptotic characteristics, such as nuclear fragmentation, appearance of apoptotic bodies and DNA laddering fragment. TUNEL assay and flow cytometric analysis also confirmed apoptosis. Furthermore, RT-PCR analysis showed that harmine induced apoptosis in B16F-10 melanoma cells by up-regulating Bax and activating Caspase-3, 9 and p53 and down-regulating Bcl-2. Harmine also up-regulated Caspase-8 and Bid, indicating that harmine affected both extrinsic and intrinsic pathways of apoptosis. This study also showed inhibitory effects of harmine on some transcription factors and pro- inflammatory cytokines that protect cell from apoptosis.

**Conclusion:**

Harmine activates both intrinsic and extrinsic pathways of apoptosis and regulates some transcription factors and pro-inflammatory cytokines.

## Background

Apoptosis, programmed cell death, occurs during normal development and tissue homeostasis or as a response to cellular insults and oncogenesis [1]. Apoptosis involves a sequence of specific morphological changes in a dying cell: condensation of the cytoplasm and nuclear chromatin, followed by breakage of cells into membrane bound apoptotic bodies containing a variety of cytoplasmic organelles and nuclear fragments, which are then engulfed by neighboring cells and macrophages [2].

Apoptosis pathways can generally be divided into signaling *via *the death receptors (extrinsic) or the mitochondria (intrinsic) pathways. Both pathways lead to activation of the members of highly selective proteases referred to as 'Caspases' [3]. A family of specific cysteine proteases ubiquitously expressed as inactive zymogens, Caspases are the key destructive molecules of apoptosis and controls all steps of apoptosis; however, in response to specific death stimuli, caspases are activated in a cascade of auto- stimulation and trans- stimulation [4]. Extrinsic pathways involve a sequential activation of Caspase-8 and 3 which cleaves target proteins, leading to apoptosis. Intrinsic pathways are directly or indirectly activated by intrinsic death stimuli such as reactive oxygen species (ROS), DNA-damaging reagents, resulting in the release of cytochrome-c and the activation of Caspase-9 which in turn activates Caspase-3 [3]. Between the death receptor and the mitochondrial signaling pathways, the pro-apoptotic protein Bid serves as a cross-talker (upon cleavage by activated Caspase-8) by inducing the translocation of the pro-apoptotic proteins Bax and/or Bak to the mitochondrial membrane [5]. The components of the extrinsic and intrinsic pathways are regulated by the members of a family of proteins called Bcl-2. Bcl-2 anti-apoptotic proteins have been targets for anticancer drug development for at least a decade [6].

P53 is a nuclear transcription factor that accumulates in response to cellular stress, including DNA damage and oncogene activation. This triggers transcriptional trans activation of p53 target genes such as p21, p27, Bax, leading to cell cycle arrest, senescence and/or apoptosis [7]. The p53 tumour-suppressor protein can intervene at every major step in apoptotic pathways as a key regulator of apoptosis and carcinogenesis [8].

Nuclear factor-κB (NF-κB) signaling pathway is generally considered as a survival factor that activates expression of various anti-apoptotic genes such as Bcl-2, Bcl-xL that block apoptosis [9]. Inhibition of NF-κB leads to down-regulation of the NF-κB-regulated anti-apoptotic proteins, thereby promoting apoptosis [3]. Expression of many pro-inflammatory cytokines is regulated at the level of transcription by the transcription factor NF-κB. Thus, inhibition of NF-κB is an important therapeutic target for the treatment of cancer [10].

Transcription factors also play a key role in controlling cell proliferation, cell cycle progression and apoptosis [11]. c-Fos and ATF-2 genes encode a nuclear transcription factor that induces transcription of a number of other genes involved in the regulation of cytokine synthesis, cell replication, cell cycle control and apoptosis. Hypophosphorylated or transcriptionally inactive forms of ATF2 reduce TNF-α expression, resulting in sensitization of melanoma to treatment *via *increased apoptosis [12-14]. In response to stress stimuli, ATF-2 activates a variety of gene targets including cyclin A, cyclin D and c-jun which are involved in oncogenesis in various tissue types [15]. Similarly cyclic AMP-response element-binding protein (CREB) was reported to suppress apoptosis, induce cell proliferation and mediate inflammation and tumour metastasis [16].

Beta-carbolines, a large group of indole alkaloids, are widely distributed in nature, such as various plants, marine creatures, insects, mammalians as well as human tissues and body fluids [17]. Harmine (7-methoxy-1-methyl-9H-pyrido [3,4-b] indole), originally isolated from the seeds of *Peganum harmala*, is a tricyclic compound belonging to the β -carboline alkaloids. These alkaloids possess a broad range of pharmacological activities, such as anxiolytic and behavioral effects [18]. Recent studies demonstrated that harmine possessed significant anti-tumor potential both *in vitro *and *in vivo *[19], *eg *significant tumor inhibition in mice bearing Lewis Lung Cancer, sarcoma180 or Hep-A tumor [20] and broad cytotoxicity spectrum against human lung carcinoma cell lines [21].

There have been no reports on the anti-proliferative and apoptotic activity of harmine on highly metastatic B16F-10 melanoma cells. Therefore, this study was conducted to explore the critical events leading to apoptosis in B16F-10 melanoma cells.

## Methods

### Cells

B16F-10 melanoma cells were obtained from National Centre for Cell Science (India). The cells were cultured in Dulbecco's Modified Eagle's Medium (DMEM) supplemented with 10% FCS (Foetal Calf Serum) and antibiotics in a humidified incubator at 37°C in 5% CO_2 _atmosphere and maintained in continuous exponential growth by twice-a-week passages.

### Chemicals and reagents

Mouse Bcl-2, Caspase-3, 8, 9, Bax, Bid, p53 and GAPDH primer sequences were obtained from Maxim Biotech (USA). Harmine was purchased from Sigma (USA). DMEM was procured from Himedia Laboratory (India). Cells-c DNA kit was purchased from Ambion (USA). Transfactor kit was purchased from BD Biosciences (USA). All other reagents used were of analytical reagent grade.

### Effects of harmine on the viability of B16F-10 melanoma cells

B16F-10 melanoma cells (5 × 10^3 ^cells/well) were plated in 96-well flat bottomed titer plate and incubated for 24 hours at 37°C in 5% CO_2 _atmosphere. Different concentrations of harmine (1-100 μg/mL) were added and incubated further for 48 hours. Before four hours of completion of incubation, 20 μl 3-4, 5-dimethylthiazol-2-yl)-2, 5-diphenyltetrazolium bromide (MTT) (5mg/mL) was added [22]. Percentage of viable cells was determined with an ELISA plate reader at 570 nm.

### Morphological analysis

B16F-10 melanoma cells (5 × 10^3 ^cells/well) suspended in DMEM were plated in 96-well flat-bottom titer plate and incubated for 24 hours at 37°C in 5% CO_2 _atmosphere. After 24 hours, various concentrations of harmine (0.5, 1 and 2 μg/mL) were added to the cells and incubated further for 48 hours under the same conditions. The cells were then washed twice with PBS (pH7.4), fixed with 5% formalin and stained with haematoxylin and eosin. The cells were observed under microscope and photographed.

### DNA fragmentation analysis

One million B16F-10 melanoma cells were treated with different concentrations of harmine (0.5, 1 and 2 μg/mL) and incubated for 24 hours at 37°C in 5% CO_2 _atmosphere. After incubation, the cells were treated with 0.1 mL lysis buffer (100 mmol/L Tris-HCl, pH8.0, containing 0.2% Triton-X100 and 1 mmol/L EDTA) for 10 minutes at -20°C. DNA was extracted according to the phenol-chloroform method [23], precipitated with chilled ethanol and re-suspended in Tris/EDTA buffer (10 mmol/L Tris-HCl, pH8.0 and 1 mmol/L EDTA). DNA samples were separated by electrophoresis in 1% agarose gels. DNA was stained with ethidium bromide and photographed under UV light.

### TUNEL assay

TUNEL assay was performed to detect apoptosis *via *DNA fragmentation by Apoptag Peroxidase *in situ *(Apoptosis detection kit, CHEMICON International, USA). B16F-10 melanoma cells (5 × 10^3 ^cells/well) suspended in DMEM supplemented with 10% FCS, 100 μg/ml streptomycin and penicillin and 2 mmol/L glutamine were plated in 96-well flat bottom titer plate and incubated for 24 hours at 37°C in 5% CO_2 _atmosphere. After 24 hours, aliquots of harmine (1 and 2 μg/mL) were added to the cells and incubated further for 48 hours under the same conditions. The cells were washed in PBS and stained according to the manufacturer's instructions. TUNEL positive cells were counted as apoptotic cells.

### Cell cycle analysis

One million B16F-10 cells suspended in DMEM were seeded in a culture flask and incubated for 48 hours at 37°C in CO_2 _atmosphere with and without harmine. Treated and untreated cells were harvested, washed with PBS and fixed with 70% ethanol for 24 hours. The cells were then centrifuged (420 × *g*, Remi, India ) and the pellet was re-suspended in PBS containing propidium idodide and RNase A. Flow cytometric analysis was performed with the FACS Calibur flow cytometer (Becton Dickinson, Singapore) using the CycleTEST PLUS DNA Reagent kit (Becton Dickinson, Singapore) according to the manufacturer's instructions.

### Effects of harmine on pro-inflammatory cytokines and GM-CSF levels

B16F-10 melanoma cells (5 × 10^3 ^cells/well) suspended in DMEM were plated in 96-well flat-bottom titer plate and incubated for 24 hours at 37°C in 5% CO_2 _atmosphere. Harmine (2 μg/mL) was added to the cells and incubated further for 48 hours under the same conditions. The supernatant was used to estimate the cytokines, namely IL-1β, IL-6, TNF-α and GM-CSF with specific ELISA kits (Pierce Biotechnology, USA) according to the manufacturer's instructions.

### Effects of harmine on gene expression

To determine the mRNA expression levels of genes responsible for triggering apoptosis, we carried out a semi-quantitative reverse transcription polymerase chain reaction (RT-PCR). B16F-10 cells were cultured with medium containing only FCS for 24 hours at 37°C in 5% CO_2 _atmosphere. Harmine (2 μg/mL per well) was added to a 96-well flat-bottom titer plate and incubated for four hours. cDNA was prepared from B16F-10 melanoma cells by cells to cDNA™ II kit (Ambion Inc, U.S.A). Briefly, cells were washed with PBS and heated in cell lysis buffer (provided in the kit) to release the RNA into the solution, followed by a heating step to inactivate endogenous RNases. The genomic DNA was further degraded by treating with DNase followed by inactivation of DNase by heating at 70°C. Reverse transcription was performed at 42°C for 50 minutes in Moloney murine leukemia virus reverse transcriptase (provided in the kit). Gene expression analysis was performed with PCR. The murine Bcl-2, Caspases-3, 8, 9, p53, Bid and Bax genes were amplified against GAPDH standard. Amplified PCR products were subjected to electrophoresis on a 1.8% agarose gel and stained with ethidium bromide and photographed under UV light.

### Effects of harmine on transcription factors

Nuclear extracts were prepared according to a previously described method [24]. B16F-10 cells suspended in serum free medium were treated with harmine for two hours at 37°C in 5% CO_2 _atmosphere. The cells were washed twice with PBS and incubated further with TNF-α (10ρg/mL) for 30 minutes to activate cytoplasmic transcription factor. The cells were then lysed with lysis buffer incubated for 15 minutes on ice. The cell suspension was centrifuged and disrupted using a syringe and centrifuged (10,000-11,000 × g, Remi,India) for 20 minutes. The crude nuclear pellet obtained is suspended in nuclear extraction buffer. Nuclei were disrupted with a fresh syringe, centrifuged and the supernatant was collected. Protein concentrations of the nuclear extracts were estimated according to the standard Bradford method and stored at -70°C.

Transcription factor profiling was performed with the BD Mercury™ Transfactor kit (BD Biosciences, USA). When nuclear extracts added to the well, DNA will bind to their consensus sequences in the well. Bound transcription factors in the DNA were detected by specific primary antibody towards NF-κBp65, NF-κBp50, NF-κB c-Rel, c-Fos, ATF-2 and CREB. A horse radish peroxidase-conjugated secondary antibody was then used to detect the bound primary antibody. The enzymatic product was measured with standard microtiter plate reader at 655 nm. Percentage inhibition was calculated according to the following formula:

where OD is optical density.

### Statistical analysis

All data were represented as mean ± standard deviation (SD). Significance levels for comparison of differences were determined with one way ANOVA, followed by Dunnet's Comparison test using Graphpad Instat (version 3.00 for Windows 98, GraphPad Software, USA). Means of the treated groups were compared with that of the control group and P < 0.05 was considered statistically significant.

## Results

### Effects of harmine on the viability of B16F-10 melanoma cells

MTT assay is a standard colorimetric assay for measuring cellular viability. MTT is reduced to purple formazan in mitochondria and is directly related to the number of viable cells. Effect of harmine on the viability of B16F-10 melanoma cells in culture is in Table 1. Harmine up to 2 μg/mL, was not directly cytotoxic to B16F-10 melanoma cells and concentrations of 0.5, 1 and 2 μg/mL were used for further experiments.

**Table 1:** Percentage cell viability of B16F-10 melanoma cells in culture after treatment with harmine

**Concentration (μg/mL) **	**Percentage of viability**
1	100
2	100
5	97.88
10	69.63
20	48.41
50	26.26
75	0
100	0
Vehicle (0.1% DMSO)	100

B16F-10 melanoma cells were incubated with different concentrations (1-100 μg/mL) of harmine. Percentage of viability was determined using MTT assay.

### Apoptotic analysis

**Figure 1:**
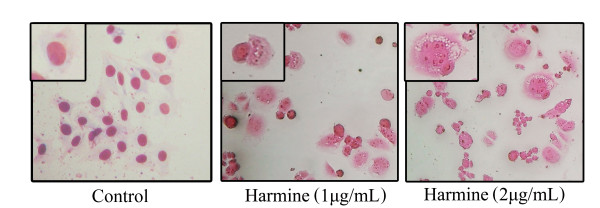
**Effect of harmine on the morphology of B16F-10 melanoma cells**. Cells treated with harmine show membrane blebbing and presence of apoptotic bodies (*n *= 3; 400×).

**Figure 2:**
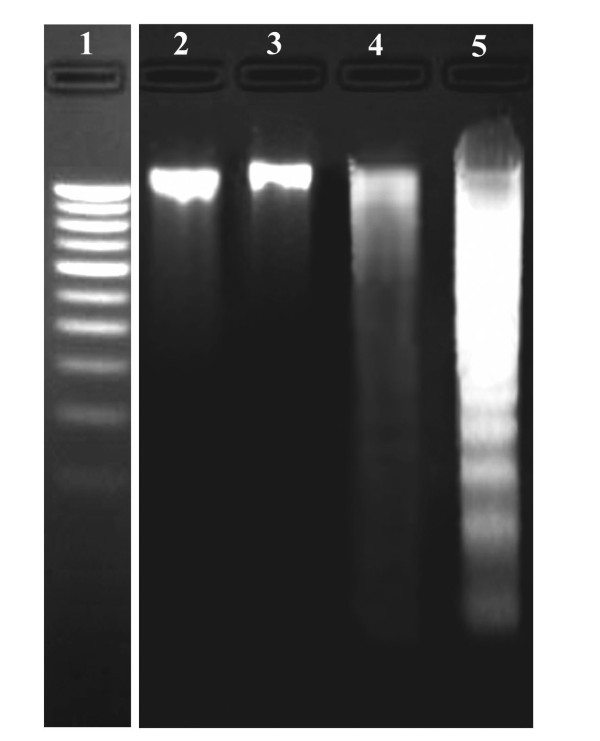
**Effect of harmine on B16F-10 melanoma DNA integrity**. Lane 1- molecular weight marker, Lane 2- DNA from untreated control cells, Lane 3-DNA from harmine (0.5 μg/mL) treated cells. Lane 4-DNA from harmine (1 μg/mL) treated cells and lane 5 -DNA from harmine (2 μg/mL) treated cells (*n *= 3).

Harmine induced marked apoptosis in B16F-10 cells. Morphological changes indicating apoptosis (*eg *membrane blebbing, chromatin condensation, DNA fragmentation, appearance of apoptotic bodies) [25] (Figure 1) were observed at 1 and 2 μg/mL of harmine by nuclear staining. The typical 'DNA ladder' was observed on DNA electrophoresis gel for treated cells at 2 μg/mL (Figure 2, lane 5). No observable changes were obtained in the morphology of cells treated with 0.5 μg/mL of harmine. Moreover, harmine at 1 and 2 μg/mL did not show any features of apoptosis on normal human umbilical vein endothelial cells (HUVEC) (data not shown).

### TUNEL assay

**Figure 3:**
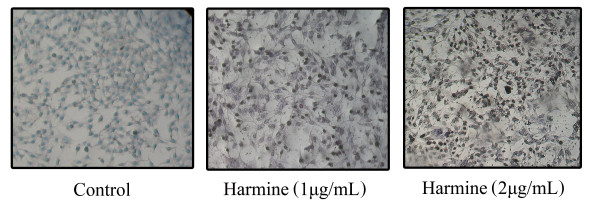
**TUNEL assay**. B16F-10 melanoma cells were treated with harmine for 48 hours and TUNEL assay was performed to detect apoptosis. TUNEL positive cells were counted as apoptotic cells. (*n *= 3; 200×).

This method is used to assay the endonuclease cleavage products by enzymatically end-labeling the DNA strand breaks [26]. Terminal transferase was used to add labeled UTP to the 3' end of the DNA fragments. As shown in figure 3, numerous TUNEL positive cells were observed when B16F-10 cells were treated with harmine at 1 and 2 μg/mL, indicating apoptotic cell death of B16F-10 melanoma cells.

### Cell cycle analysis

**Figure 4:**
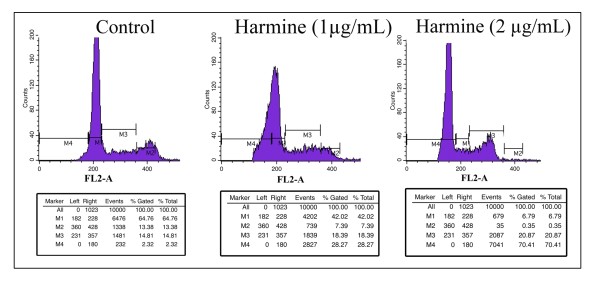
**Effect of harmine on cell cycle progression**. B16F-10 melanoma cells were treated with harmine for 48 h and analyzed for propidium iodide stained-DNA content by flow cytometry. Values indicate the percentage of the cell population at the phase of the cell cycle. M_1 _= G_1 _(Diploid), M_2 _= G_2_/M (Tetraploid), M_3 _= S (Synthetic phase), M_4 _= Sub-G_1 _phase. The population of cells in the sub-G_0_/G_1 _phase represents cellular fragments due to apoptosis.

The effects of the harmine on cell cycle distribution were determined (Figure 4). Harmine inhibited cell growth with arrest at G_1 _and reduced transition to the S and G_2_/M phases of the cell cycle. The proportion of the sub-G_0_/G_1 _peak was negligible in the control (2.32%) cells and most cells (79.57%) were in G1 and S phases due to the high proliferative state of B16F-10 cell line. Exposure of cells to harmine (1 and 2 μg/mL) for 48 hours resulted in cell accumulation at the sub-G_0_/G_1 _phase in a dose-dependent manner. At 1 μg/mL 28.27% cells were accumulated and 70.41% cells at 2 μg/mL.

### Effects of harmine on pro-inflammatory cytokine and GM-CSF levels

Harmine significantly inhibited the production of pro-inflammatory cytokines, namely TNF-α, IL-1β, IL-6 and GM-CSF by B16F-10 melanoma cell in culture (Table 2). Harmine (2 μg/mL) showed maximum inhibition of all cytokines.

**Table 2:** Effect of harmine on the release of TNF-α, IL-1β, IL-6 and GM CSF by B16F-10 melanoma cells

Cytokine (pg/mL)		Harmine	
	Control	1 μg/mL	2 μg/mL
TNF-α	29.17 ± 5.94	26.18 ± 1.71	20.19 ± 1.12 (*P *= 0.012)
IL-1β	98.15 ± 2.08	74.48 ± 2.11 (*P *< 0.001)	61.34 ± 2.34 (*P *< 0.001)
IL-6	61.43 ± 4.44	40.42 ± 5.19 (*P *< 0.001)	28.46 ± 2.80 (*P *< 0.001)
GM-CSF	34.55 ± 1.07	29.55 ± 1.21 (*P *= 0.01)	22.81 ± 1.19 (*P *< 0.001)

B16F-10 cells (5 × 10^3 ^cells) were cultured in the presence of harmine for 48 hours, and level of pro-inflammatory cytokines in the culture supernatant was estimated. Values are expressed as mean ± SD. Statistical analysis was performed with ANOVA, followed by Dunnet's test using GraphPad Instat software.

### Effects of harmine on gene expression

**Figure 5:**
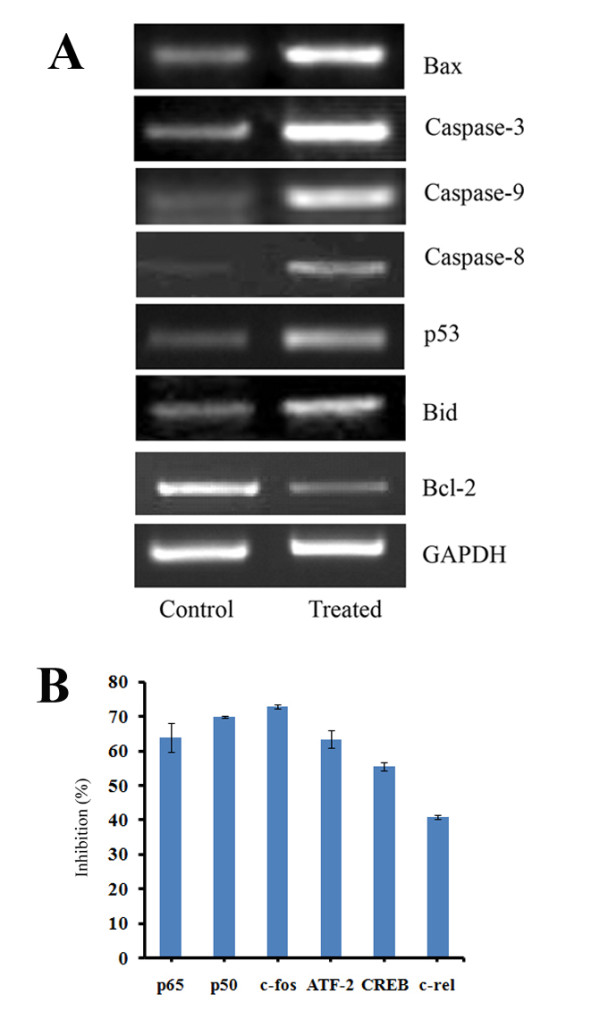
**(A) Effect of harmine on gene expression**. B16F-10 cells were cultured in the presence and absence of harmine (2 μg/mL) and cDNA was synthesized and amplified with appropriate primers using PCR. The mRNA expression levels were normalized with GAPDH (house-keeping gene). (B) Effect of harmine on transcription factors. B16F-10 melanoma cells were treated with harmine (2 μg/mL) for two hours and then incubated with TNF-α (10 pg/mL) for 30 minutes. Percentage inhibition in various transcription factors were measured by ELISA method.

RT-PCR analysis revealed a significant down regulation in the expression of Bcl-2 gene compared to control. At the same time, expression of pro-apoptotic genes such as p53, Caspase-3, 8, 9, Bid and Bax were significantly up-regulated by the treatment with harmine, which indicated the involvement of harmine in both intrinsic and extrinsic pathways of apoptosis. Cell death mechanism induced by the harmine in B16F-10 melanoma cells may be mediated by the activation of these genes controlling both intrinsic and extrinsic pathways of apoptosis (Figure 5A).

### Effects of harmine on transcription factors

The DNA bound transcription factor was determined with corresponding primary antibody, which was detected with horseradish peroxidase-conjugated secondary antibody. The percentage inhibition in the activation/translocation NF-κB sub units, namely p65, p50 and c-Rel, were 64.07, 70.08 and 41.03 respectively after harmine treatment with. Inhibition in the activation of other transcription factors such as c-Fos (73.11%), ATF-2 (63.51%) and CREB (55.59%) were also observed with harmine treatment (Figure 5B).

## Discussion

In the present study, treatment of melanoma cells with harmine induced morphological changes including condensation of nuclear chromatin, formation of apoptotic bodies and blebbing of the cell membrane. All these morphological characteristics are biochemical hallmarks of apoptosis, indicating that apoptosis may play a crucial role in cell death elicited by the harmine on B16F-10 melanoma cells. DNA extracts from harmine treated B16F-10 melanoma cells also showed characteristic ladder pattern of discontinuous DNA fragments. Moreover, presence of pyknotic nuclei (characteristic of cells undergoing apoptosis [27] was further confirmed with tunnel assay. MTT assay ruled out necrosis as a probable cause of cell death in harmine treated cells as most of the cells exhibited intact plasma membranes.

P53 is a nuclear transcription factor that accumulates in response to cellular stress, including DNA damage and oncogene activation. This triggers transcriptional transactivation of p53 target genes such as p21, Bax, leading to cell cycle arrest, senescence and/or apoptosis [7]. The mitochondrial death pathway is controlled by members of the Bcl-2 family, including the anti-apoptotic Bcl-2 and the pro-apoptotic Bax and Bid proteins. The pro-apoptotic Bcl-2 family members Bax is crucial in regulating a wide range of apoptotic stimuli [28] and become activated by Bcl-2 family members that have only the BH3 domain, namely Bid [29]. It was reported that over expression of Bax results in the release of cytochrome- c from mitochondria to the cytosol and induction of apoptosis [30] and that the direct incubation of Bax protein with isolated mitochondria also induced cytochrome-c release [31]. P53 is a potent activator of the caspase cascade by stimulating pro-apoptotic proteins (Bid and Bax) and promoting the release of apoptogenic factors (cytochrome c), leading to Caspase-9 activation and in turn cleaving effector caspases such as Caspase-3 [32]. Expression analysis of mRNA revealed the apoptotic regulation of various genes in B16F-10 melanoma cells treated with harmine. Expression of pro-apoptotic genes such as P53, Caspase-3, 8 and 9, Bid, Bax was significantly induced at the earlier phase of treatment (4 hours), suggesting that harmine was an initiator or inducer of the apoptotic mechanism. Harmine could enhance the activation of Bcl-2 family pro-apoptotic proteins such as Bax and Bid while it could also down-regulate the expressions of Bcl-2 in B16F-10 melanoma cells. Activation of Caspase-8 and Bid along with other caspases indicates the involvement of harmine in both extrinsic and intrinsic pathways of apoptosis because Bid serves as a cross-talker upon cleavage by activated Caspase-8 by inducing the translocation of the pro-apoptotic proteins Bax and/or Bak to the mitochondrial membrane [5]. Tumor apoptosis was closely associated with its cell cycle arrest. Over expression of cyclin dependent kinase inhibitors such as p27, p21 may lead to apoptosis of tumor cells, inhibit their proliferation and diminish their metastasis [33,34]. The present study found that harmine caused cell cycle arrest in G0/G1 phase and showed an evident apoptotic sub-G0/G1 peak in B16F10 melanoma cells.

The NF-κB protein family encompasses transcription factors involved in controlling the expressions of genes crucial for several important cellular signal transduction pathways in inflammation, proliferation and in defense against apoptosis. Constitutive activation of NF-κB and chronic inflammation has a major role in the development of most tumors, including leukemia, lymphomas and solid tumours. Inhibition of NF-κB leads to down-regulation of the NF-κB-regulated anti-apoptotic proteins and other pro-inflammatory cytokines, thereby promoting apoptotic cell death [35,36]. In this study, inhibition of the activation of NF-κB was probably attributed to the decreased production of pro-inflammatory cytokines in B16F10 melanoma cells.

Genes controlling transcription is deregulated in a wide range of cancers; thus, targeting proteins that regulate signaling pathways for translation and protein synthesis is a realistic strategy for cancer treatment. Members of the AP-1 (activator protein-1) family are necessary for cell cycle progression in several cell systems and also for cell transformation induced by a variety of oncogenes, including Src, Ras and Raf [37]. ATF-2 regulates the transcription of several genes involved in cytokine synthesis, cell cycle control apoptosis and DNA repair [38]. Cyclin D1, an important gene for the integration of proliferative and anti-proliferative signals during the G1 phase of the cell cycle, possesses a CRE element within its promoter region. In murine chondrocytes, cyclin D1 is directly activated by ATF-2 while the levels of activation are reduced in ATF-2-deficient mice. Cyclin D1 is activated by ATF-2 in proliferating murine melanoma cells [14]. CREB also regulates the expression of a repertoire of genes related to cell survival, inflammation and proliferation, such as Bcl-2, Bcl-xL, COX-2 and TNF-α [15]. As these transcription factors are major negative regulators of apoptosis, their inhibition by harmine promotes apoptosis in B16F-10 melanoma cells.

## Conclusion

Harmine activates both intrinsic and extrinsic pathways of apoptosis and regulates some transcription factors and pro-inflammatory cytokines.

## Abbreviations

Bax: Bcl-2 associated X protein; Bid: BH3 interacting domain death agonist; CREB: cyclic AMP-response element-binding protein; DMEM: Dulbecco's Modified Eagle's Medium; FCS: Foetal Calf Serum; GM-CSF: Granulocyte monocyte colony stimulating factor; IL: Interleukin; MTT: 3-4, 5-dimethylthiazol-2-yl)-2, 5-diphenyltetrazolium bromide; NF: Nuclear factor; ROS: Reactive oxygen species; TNF: Tumour necrosis factor; TUNEL: Terminal deoxynucleotidyl transferase dUTP nick end labeling

## Competing interests

The authors declare that they have no competing interests.

## Authors' contributions

GK designed and coordinated the study. TPH carried out the study including acquisition, analysis and interpretation of the data. Both authors read and approved the final version of the manuscript.
